# Contact pressure distribution of the hip joint during closed reduction of developmental dysplasia of the hip: a patient-specific finite element analysis

**DOI:** 10.1186/s12891-020-03602-w

**Published:** 2020-09-08

**Authors:** Zhiqiang Zhang, Dashan Sui, Haiyi Qin, Hai Li, Ziming Zhang

**Affiliations:** 1 Department of Orthopedics, National Children’s Medical Center & Children’s Hospital of Fudan University, 399 Wanyuan Road, Shanghai, 201102 China; 2 School of Materials Science and Engineering, Shanghai Jiao Tong University, 1954 Huashan Rd, Shanghai, 200030 China; 3 Department of Pediatric Orthopedics, Xin Hua Hospital Affiliated to Shanghai Jiao Tong University School of Medicine, 1665 Kongjiang Road, Shanghai, 200092 China

**Keywords:** Developmental dysplasia of the hip, Closed reduction, Finite element analysis, Cartilage contact pressure, Avascular necrosis

## Abstract

**Background:**

Developmental dysplasia of the hip (DDH) is the most common deformity of the lower extremity in children. The biomechanical change during closed reduction (CR) focused on cartilage contact pressure (CCP) has not been studied. Thereby, we try to provide insight into biomechanical factors potentially responsible for the success of CR treatment sand complications by using finite element analysis (FEA) for the first time.

**Methods:**

Finite element models of one patient with DDH were established based on the data of MRI scan on which cartilage contact pressure was measured. During CR, CCP between the femoral head and acetabulum in different abduction and flexion angles were tested to estimate the efficacy and potential risk factors of avascular necrosis (AVN) following CR.

**Results:**

A 3D reconstruction by the FEA method was performed on a 16 months of age girl with DDH on the right side. The acetabulum of the involved side showed a long, narrow, and “flat-shaped” deformity, whereas the femoral head was smaller and irregular compared with the contralateral side. With increased abduction angle, the stress of the posterior acetabulum increased significantly, and the stress on the lateral part of the femoral head increased as well. The changes of CCP in the superior acetabulum were not apparent during CR. There were no detectable differences in terms of pressure on the femoral head.

**Conclusions:**

Severe dislocation (IHDI grade III and IV) in children showed a high mismatch between the femoral head and acetabulum. Increased abduction angle corresponded with high contact pressure, which might relate to AVN, whereas increased flexion angle was not. Enhanced pressure on the lateral part of the femoral head might increase the risk of AVN.

## Background

Developmental dysplasia of the hip (DDH) is the most common developmental malformation affecting children’s hips. The principle of treatment is to establish a stable, concentric reduction of the hip to enable the subsequent hip development as early as possible, given the well-established correlation between residual dysplasia and the age of reduction [1, 2].

The mutual stimulation of the femoral head and the acetabulum adapts to the physiological and biomechanical demands of reciprocal growth and development [3]. Closed reduction (CR) of the hip is indicated in patients who failed to achieve stable reduction with Pavlik harness, and or as the primary treatment option for patients with late diagnosis [4, 5].

Finite element analysis (FEA) is especially useful in bioengineering and biomechanical modeling. FEA can replace biomechanical experiments to some extent, and can control the experimental conditions and simulate the biomechanical conditions of the human body [6].

This study aimed to provide insight to identify biomechanical factors potentially responsible for the success of CR or post-treatment complications by using finite element analysis (FEA). Therefore, we evaluated the stress changes of bone and cartilage during CR and tried to answer the following questions: 1. Why does acetabular index (AI) have a high reference value to predict the failure of CR? 2. Why does higher International Hip Dysplasia Institute (IHDI) grade correlate to higher failure rate? 3. Why does extreme reduction increase stability but also increases the risk of avascular necrosis (AVN)?

## Methods

### Subject clinical information

**Fig. 1:**
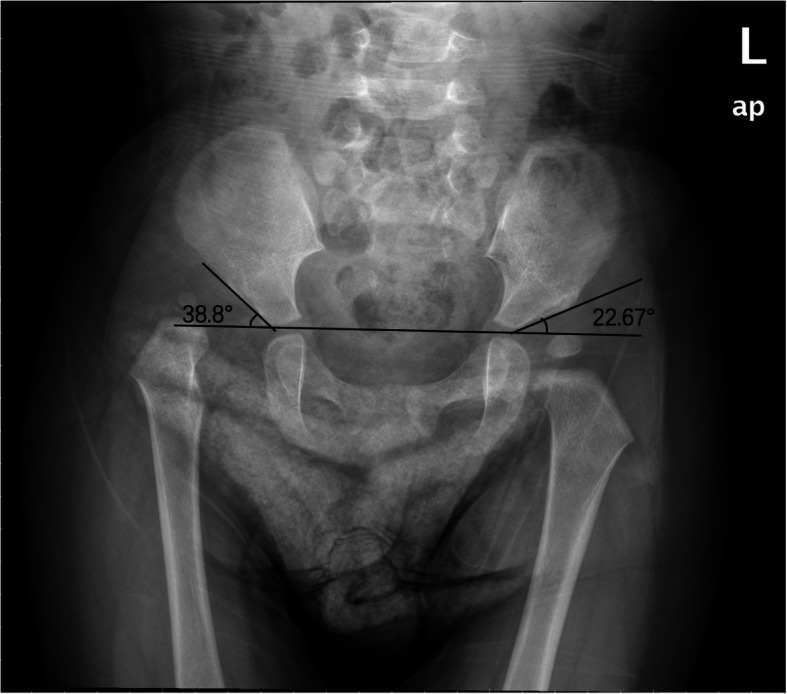
X-ray of the DDH patient. Anteroposterior X-ray showed the subject (16 months old girl) with right dislocation of the hip. The AI was 38.8°on the right side and 22.67° on the left side, respectively

After the parents and institution’s Ethics Committee approval, a pediatric DDH patient was randomly selected as the subject for this study in our hospital, who was a female of 16 months of age, diagnosed with unilateral DDH of the right hip. The patient is 80 cm tall, weighs 12.6 kg. Physical examinations showed that she had a Trendelenburg gait and limb length discrepancy of 1 cm shorter on the right side and positive Allis sign. Radiographs showed large acetabular index (AI) (38.80°) and was classified as grade IV of IHDI on the right side; The AI of the left side was 22.67° (Fig. 1).

### Closed reduction

CR under fluoroscopic guidance was performed under general anesthesia. The hip was reduced by placing it in flexion nearly 100 degrees and gradually abducting it on the position of stability. Then a hip spica cast was fixed in a human position with a gentle posterior mold.

### Imaging

To create patient-specific anatomic models, MRI was used to capture nonbony geometry. On a Siemens 3.0 T symphony MR scanner, we obtained coronal 3D gradient-echo images of the pelvis and proximal femur, slice thickness 2.6 mm, matrix 640*640, and 10 min scan time.

### Model assembly

First, the DICOM data were imported into the Mimics software (17.0, Materialise). Next, different masks were set up by the processes of thresholding, region growing, manual editing, Boolean operation, etc. Finally, the 3D models of four bones and three cartilages were established based on these masks.

### FEA modeling

**Fig. 2:**
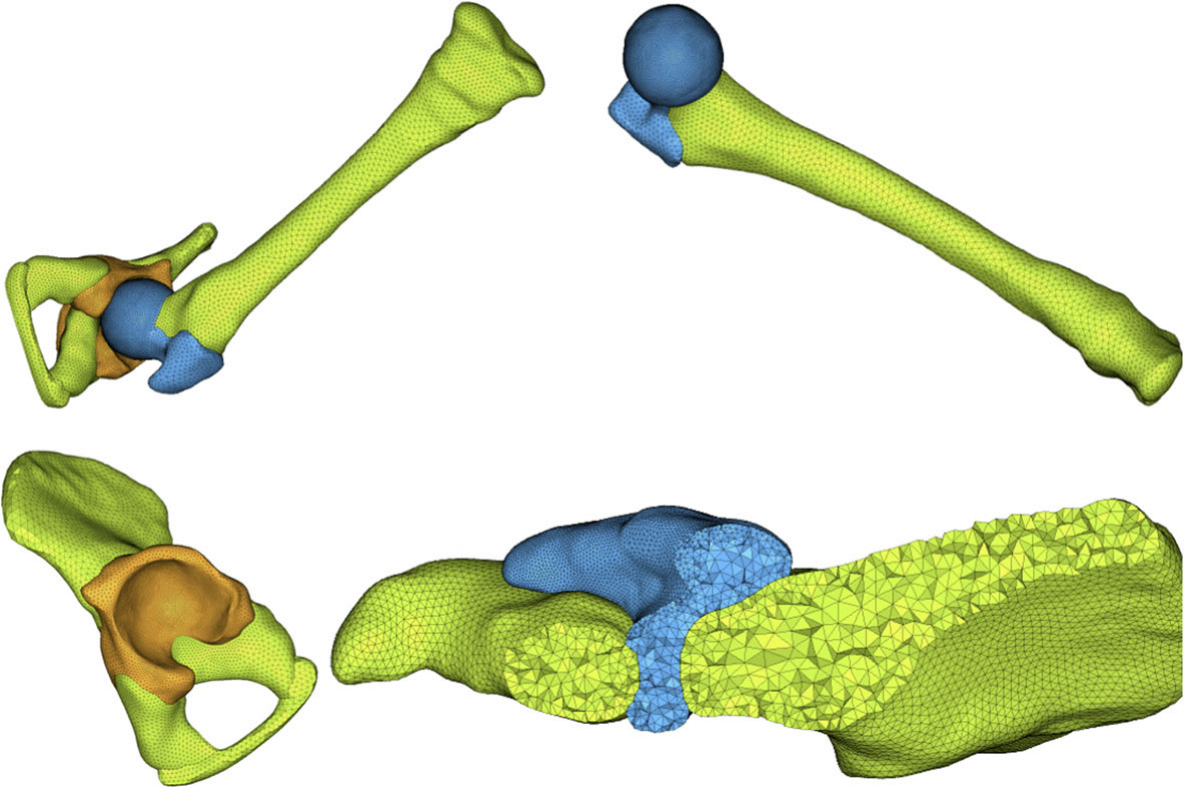
The CAD and FEA model of hip. The type of tetrahedral solid element was used in the analysis, and there are 228,000 tetrahedral elements and 49,000 nodes in the FEA model

The CAD models were imported into the software, Hyper-mesh (13.0 Altair), and the FEA models were established by element size and local refinement based on the requirement of mechanical analysis, and the acetabulum was divided into six regions. (AL: Anterolateral; AM: Anteromedial; SL: Superolateral; SM: Superomedial; PL: Posterolateral; PM: Posteromedial.) (Supplementary Fig. 1) The type of tetrahedral solid element was used in the analysis, and there are 228,000 tetrahedral elements and 49,000 nodes in the FEA model. In order to improve the convergence of contact calculation, the elements on the contact surfaces of the femoral head and acetabulum were refined. Moreover, the selective reduction integral with node rotation was adopted for tetrahedral elements to improve the convergence of the solution preferably (Fig. 2).

### Mechanical properties of materials

**Fig. 3:**
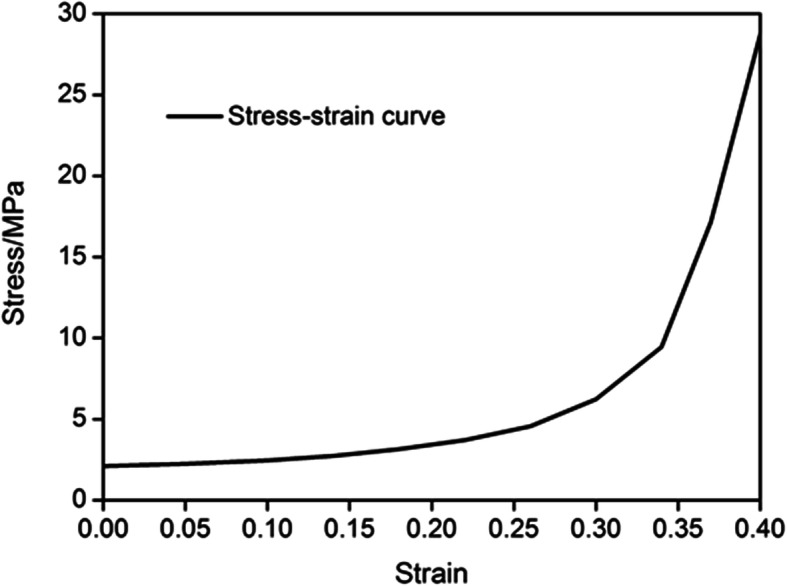
The stress-strain curve of cartilage. The properties of cartilage are not exactly identical from surface to inside. As the strain increases, the stress increases exponentially

There were two materials, namely bone and cartilage, in the FEA model [7]. The young’s modulus was defined as 17GPa, and the Poisson ratio was 0.3 for the cortical bone. The cartilage was a viscoelasticity material that its mechanical features were influenced by different sclerostin and loading rate. Moreover, according to different bone age, properties of cartilage are also not exactly identical from surface to inside. Thus, the cartilage was described as an inhomogeneous material. It was difficult to describe the mechanical properties of cartilage accurately by using a mathematical curve. The stress-strain curve was defined approximately according to the previous research [8] (Fig. 3).

### Definition of boundary conditions

When a relaxed muscle is stretched beyond its resting length, it behaves as a deformable body: it deforms and provides passive resistance to the stretch, and the passive response is characterized as hyperplastic. Under the conditions of leg abduction and flexion, the adductor muscles are the main resistance. The points of attachment and physiological cross-sectional area (PCSA) were determined for the seven muscles (Pectinus, Adductor Magnus 1, Adductor brevis, Adductor longus, Adductor Magnus 2, Adductor Magnus 3, Gracilis), respectively, according to the reference [9]. Given the individual difference of the PCSA among patients, the PCSA of a muscle in a subject was defined as a ratio value of one to the whole, according to described in literature [10].

Step 1, Based on the fact that the muscle’s force is proportional to its PCSA. The relationship between force and PCSA is F = k*A (1). The k is an unknow constant.

Step 2, Given the difference of the PCSA among children and adults, the PCSA of a muscle in a subject was defined as a ratio value of know PCSA described in literature [10]. A = PCSA*α (2). (Table 1) The α is another unknow constant.

**Table 1:** The relationship of length and PCSA under different flexion angle for different muscle

Number	Muscles	Friederich PCSA (mm^2^)	FA 0° PCSA (mm^2^)	FA 0° ML (mm)	FA 45° ML (mm)	FA 65° ML (mm)	FA 80° ML (mm)
1	Pectinus	903	A1 = 903*α	31.5	49.1	55.7	59.3
2	Adductor Magnus (minimus)	2552	A2 = 2552*α	63.3	88.5	96.0	100.2
3	Adductor brevis	1152	A3 = 1152*α	72.4	97.2	104.5	108.7
4	Adductor longus	2273	A4 = 2273*α	93.5	121.5	130.8	135.4
5	Adductor Magnus (middle)	1835	A5 = 1835*α	111.3	134.9	141.6	146.1
6	Adductor Magnus (posterior)	1695	A6 = 1695*α	134.8	161.7	170.4	175.8
7	Gracilis	373	A7 = 373*α	137.4	164.0	172.9	177.9

*PCSA* physiologic cross sectional area [11], *FA* flexion angle, *ML* muscle length, a: a unknown coefficient, which can be used to predict the new PCSA, according to the Friederich PCSA

Step 3, Expert radiologist judgment and assessment of a 3D FEA model were employed to finalize attachment locations of each muscles. Then, we calculated *the length of each muscles* (L) according to the points of attachment.

Step 4, Supposedly muscles were in the natural state when the thigh was in the state of 90-degree flexion and 0-degree abduction. At this position, the length of the muscle was defined as L_a1_, and the PCSA was A_a1_.

Combined with formula (1) (2).

$$ {\displaystyle \begin{array}{l}{\mathrm{F}}_{1\mathrm{a}}=\mathrm{k}\ast \upalpha \ast {\mathrm{PCSA}}_{1\mathrm{a}}\\ {}\dots \\ {}{\mathrm{F}}_{7\mathrm{a}}=\mathrm{k}\ast \upalpha \ast {\mathrm{PCSA}}_{2\mathrm{a}}\end{array}} $$

We assumed that the total muscle force equals 1.

Then F_Ta_=F_1a_+F_2a_+ … +F_7a_=1

$$ {\displaystyle \begin{array}{l}{\mathrm{F}}_{1\mathrm{a}}/{\mathrm{F}}_{\mathrm{Ta}}={\mathrm{PCSA}}_{1\mathrm{a}}/\left({\mathrm{PCSA}}_{1\mathrm{a}}+{\mathrm{PCSA}}_{2\mathrm{a}}+\dots +{\mathrm{PCSA}}_{7\mathrm{a}}\right)=0.083\\ {}\dots \\ {}{\mathrm{F}}_{7\mathrm{a}}/{\mathrm{F}}_{\mathrm{Ta}}=0.035\end{array}} $$

Step 5, The total volume remains constant when the muscle abducts from angle a to angle b.

3 $$ {\mathrm{L}}_{1\mathrm{a}}\ast {\mathrm{A}}_{1\mathrm{a}}={\mathrm{L}}_{1\mathrm{b}}\ast {\mathrm{A}}_{1\mathrm{b}} $$

Combined with formula (1) (2) (3).

$$ {\displaystyle \begin{array}{l}{\mathrm{F}}_{1\mathrm{b}}=\mathrm{k}\ast {\mathrm{A}}_{1\mathrm{b}}=\mathrm{k}\ast {\mathrm{A}}_{1\mathrm{a}}\ast {\mathrm{L}}_{1\mathrm{a}}/{\mathrm{L}}_{1\mathrm{b}}=\mathrm{k}\ast \mathrm{a}\ast {\mathrm{PCSA}}_{1\mathrm{a}}\ast {\mathrm{L}}_{1\mathrm{a}}/{\mathrm{L}}_{1\mathrm{b}}\\ {}{\mathrm{F}}_{2\mathrm{b}}=\mathrm{k}\ast \upalpha \ast {\mathrm{PCSA}}_{2\mathrm{a}}\ast {\mathrm{L}}_{2\mathrm{a}}/{\mathrm{L}}_{2\mathrm{b}}\\ {}\dots \\ {}{\mathrm{F}}_{7\mathrm{b}}=\mathrm{k}\ast \upalpha \ast {\mathrm{PCSA}}_{7\mathrm{a}}\ast {\mathrm{L}}_{6\mathrm{a}}/{\mathrm{L}}_{7\mathrm{b}}\end{array}} $$

We assumed that the total muscle force equals 1.

Then F_Tb_=F_1b_+F_2b_+ … +F_7b_=1

$$ {\displaystyle \begin{array}{l}{\mathrm{F}}_{1\mathrm{b}}/{\mathrm{F}}_{\mathrm{Tb}}={\mathrm{PCSA}}_{1\mathrm{a}}\ast {\mathrm{L}}_{1\mathrm{a}}/{\mathrm{L}}_{1\mathrm{b}}/\left({\mathrm{PCSA}}_{1\mathrm{a}}\ast {\mathrm{L}}_{1\mathrm{a}}/{\mathrm{L}}_{1\mathrm{b}}+{\mathrm{PCSA}}_{2\mathrm{a}}\ast {\mathrm{L}}_{2\mathrm{a}}/{\mathrm{L}}_{2\mathrm{b}}+\dots +{\mathrm{PCSA}}_{7\mathrm{a}}\ast {\mathrm{L}}_{7\mathrm{a}}/{\mathrm{L}}_{7\mathrm{b}}\right)=0.070\ \left(\mathrm{For}\ \mathrm{example}\ \mathrm{b}=45{}^{\circ}\right).\\ {}\dots \end{array}} $$

F_7b_/F_Tb_ = 0.038 (Table 2).

**Table 2:** The ratio of muscle force under different abdution angle for different muscle

Number	Muscles	AA 0° TRMF	AA 45° TRMF	AA 65° TRMF	AA 80° TRMF
1	Pectinus	0.083	0.07	0.066	0.065
2	Adductor Magnus (minimus)	0.237	0.221	0.219	0.217
3	Adductor brevis	0.107	0.104	0.104	0.103
4	Adductor longus	0.211	0.212	0.211	0.212
5	Adductor Magnus (middle)	0.170	0.183	0.187	0.189
6	Adductor Magnus (posterior)	0.157	0.171	0.174	0.175
7	Gracilis	0.035	0.038	0.039	0.039
Total	1	1	1	1	1

*AA* abduction angle, *TRMF* The ratios of muscle forces

Step 6, The abduction force was defined approximately as 1/5 of the baby weight (24 N) [12]. Based on the FEA model, the value of the force for each muscle can be solved by force analysis according to the force ratio of each muscle when the muscle forces and external applied loads staying in the equilibrium state.

### Mechanical analysis

**Fig. 4:**
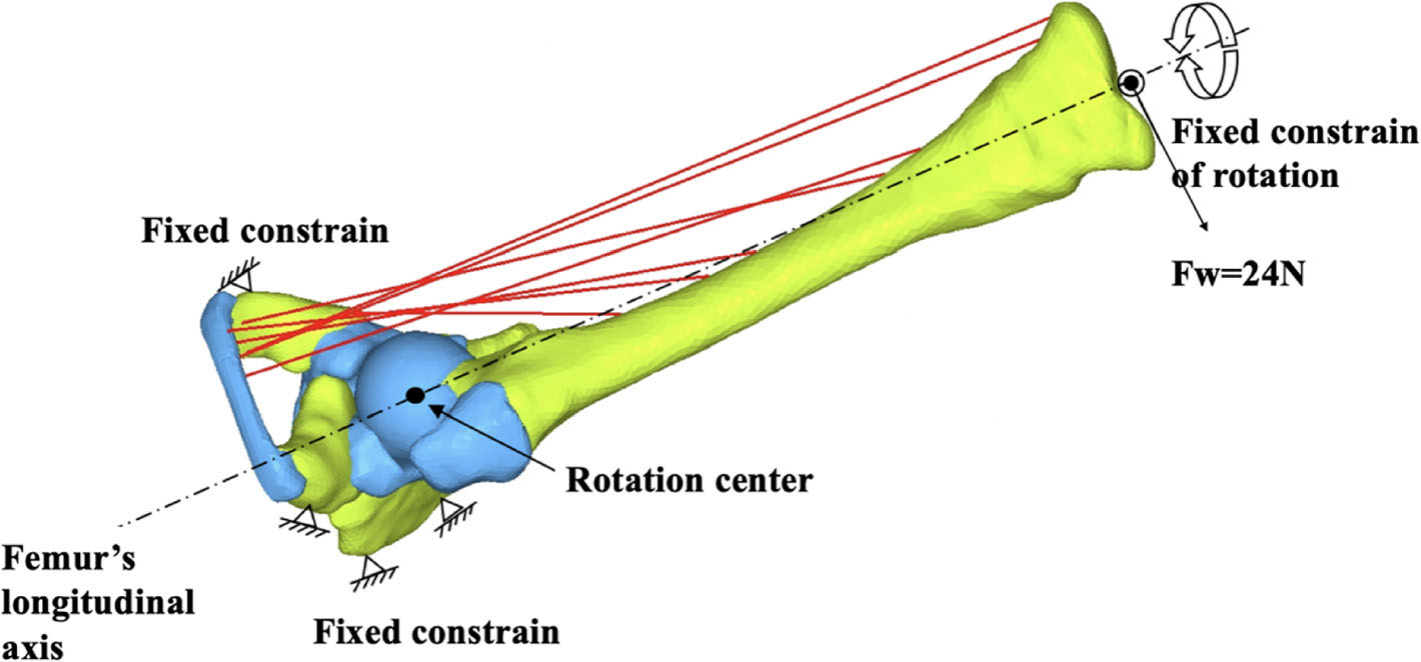
Force analysis during CR. The abduction force, Fw, was applied on the point of knee, with the force direction being vertical to the axis of the thigh. The fixed constraint was applied to the surrounding of the femur and to the rotation of thigh axis to simulate the restriction of surrounding tissues. The value of the force for each muscle can be solved according to the table of force ratio of each muscle when the muscle forces and external applied loads staying in the idle state for the thigh

To simulate the action of CR, the abduction force, F, was applied on the point of knee, with the force direction being vertical to the axis of the thigh. The fixed constraint was applied to the surrounding of the femur and to the rotation of thigh axis to simulate the restriction of surrounding tissues. A muscle produces two kinds of forces, active and passive, which sum to compose muscle total force. Under the condition of leg abduction and flexion, the adductor muscles stretched beyond its resting length are the main resistance of the passive response. Thus, the active force is neglected. The abduction force was defined approximately as 1/5 of the baby weight (24 N) [12]. Based on the FEA model, the value of the force for each muscle can be solved according to the force ratio of each muscle when the muscle forces and external applied loads (24 N) staying in the equilibrium state. Moreover, the distribution of acting force and stress between the femoral head and the acetabulum also could be solved (Fig. 4).

## Results

**Fig. 5:**
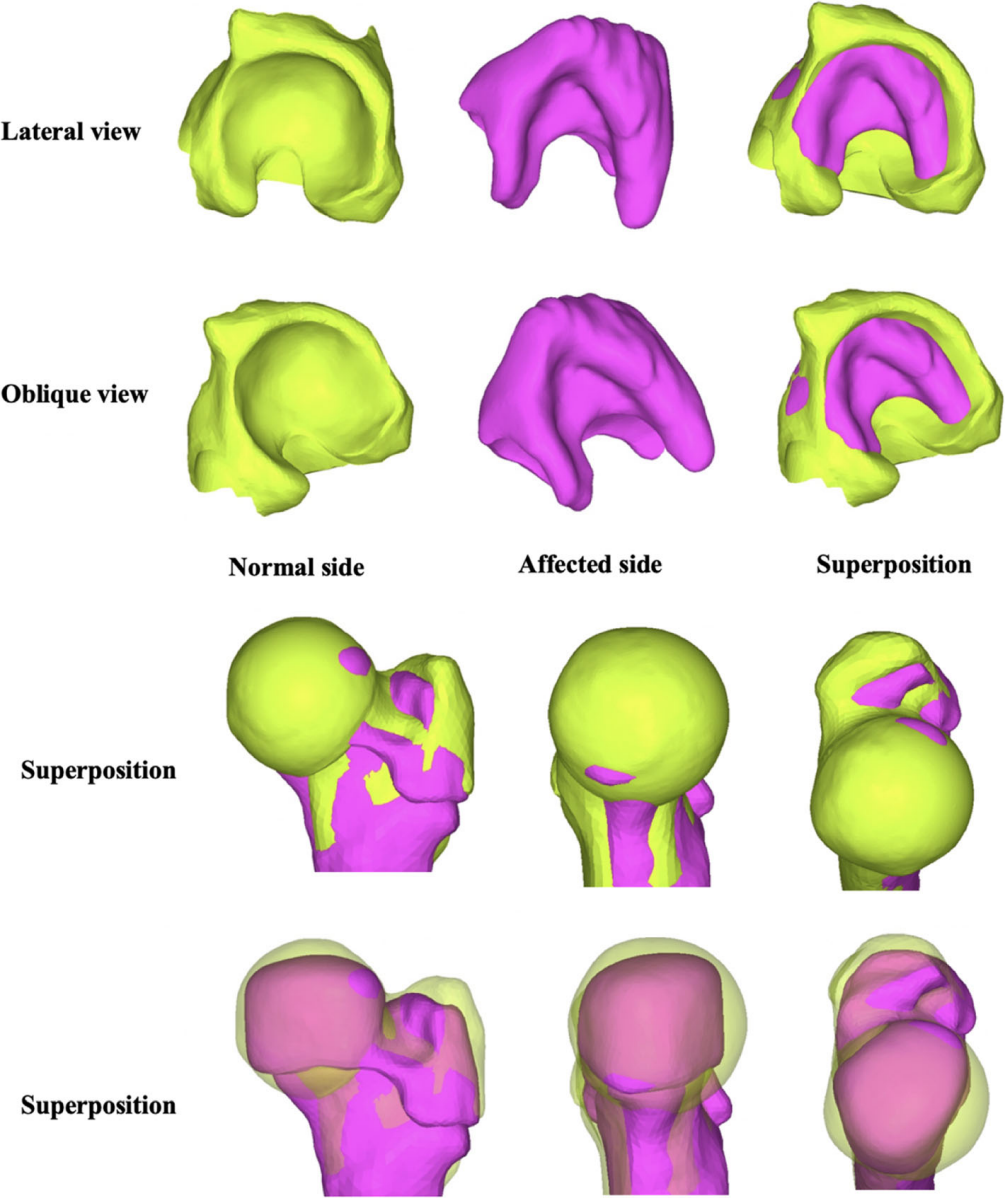
Morphology of the normal side and affected side of the acetabulum and the proximal femur. Through the construction of the cartilage model of hip, it perfectly showed us the “flat-shaped” deformity of the affected side (purple) as compared with the “cup-shaped” acetabulum of the normal side (light green)

Compared with the healthy side, the dislocated side was manifested with long and narrow malformation of acetabulum. Due to the lack of the femoral head stimulation, the acetabulum was not deep enough, showing a “flat” shape. The corresponding affected femoral head also had an irregular shape, which further increased the difficulty of matching the acetabulum when performing CR. (Fig. 5).

Among the seven adductor muscles, adductor magnus minimus and adductor longus had the most massive muscle force, while the pectineus, adductor brevis and gracilis had the least force. Larger muscular force was combined with larger abduction angles, and the fact that abduction angle increased from 65 to 80 degrees was dramatically more significant than the increase from 45 to 65 degrees (Supplementary Fig. 2).

**Fig. 6:**
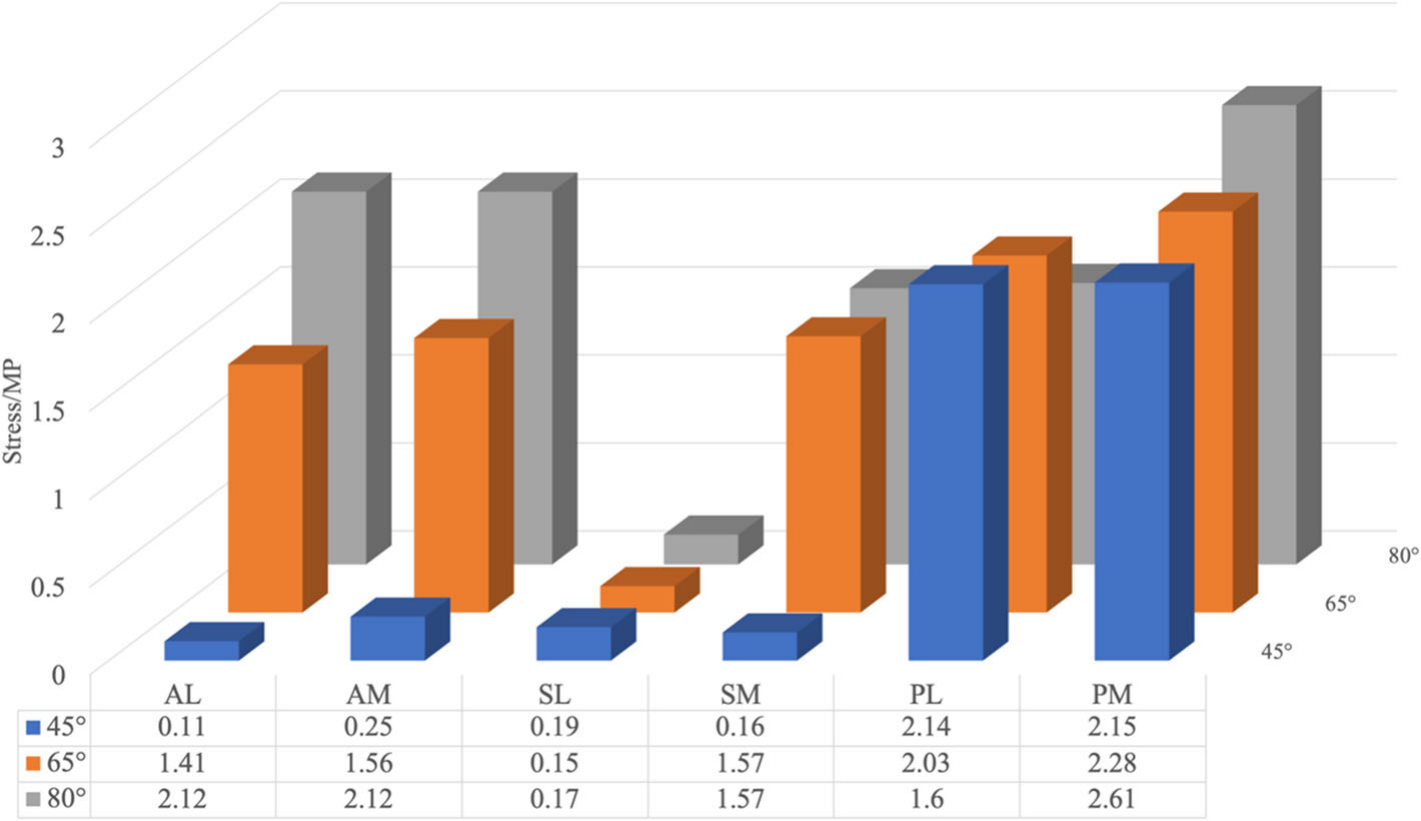
Stress distribution of different areas of acetabulum under various abduction angles. When the hip was flexed at 90° and abducted at 45°, the stress was mainly concentrated posteriorly. With the angle increased to 65°, the stress on the anterior area increased correspondingly, but the stress on the posterior had not increased significantly, and distribute the pressure over the contact area more uniformly between the femoral head and the u-shaped notch, without excessive pressure on the femoral head. However, when the abduction of the hip was increased to 80°, CCP on the anterior and posteromedial acetabulum increased significantly, and the corresponding anterolateral and lateral stresses of the femoral head increased significantly

**Fig. 7:**
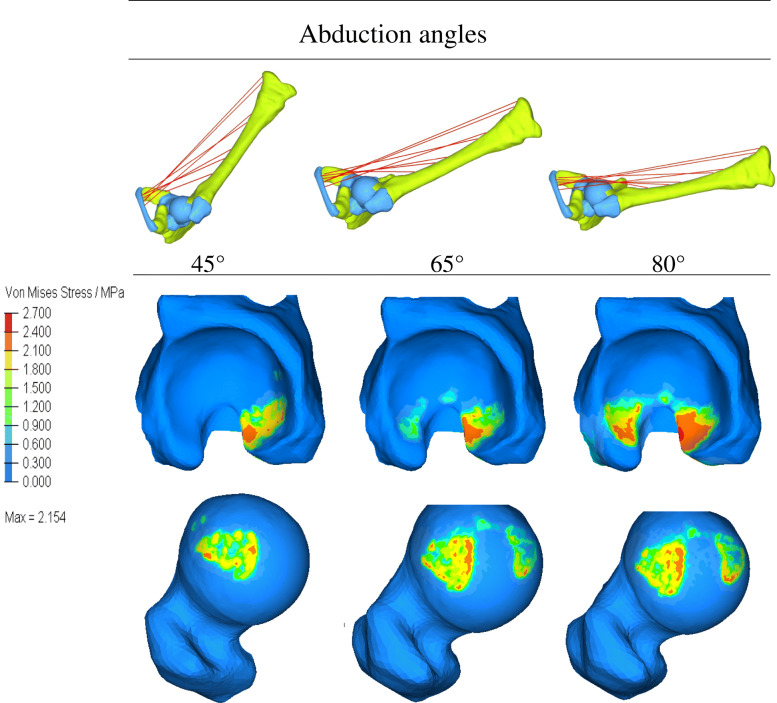
Distributions of CCP on the acetabular surface and the femoral head for various abduction angles. The contact area between the acetabular and the femoral head was larger on anterior and posterior than superior area, and with the increase of abduction angle, CCP became more and more uniform. Extreme abduction angle (such as 80°) would significantly increase CCP on acetabulum in both front and behind, and both the corresponding anteromedial and lateral stress of the femoral head would substantially increase

**Fig. 8:**
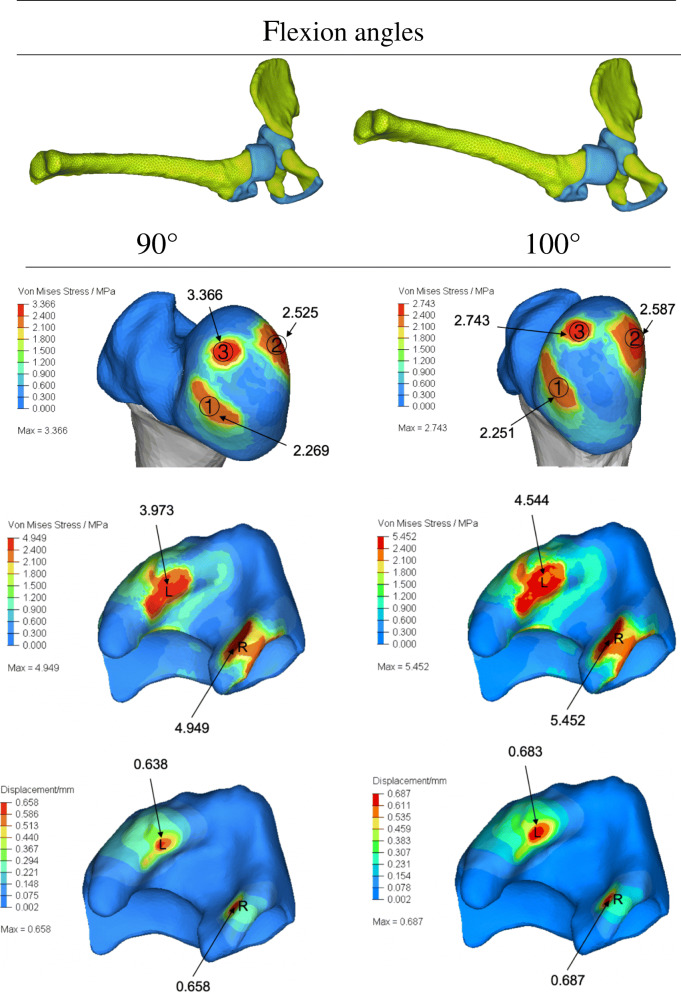
Distributions of CCP on the acetabular surface and the femoral head and the cartilage displacement of acetabulum for various flexion angles. Compared with 90 degrees flexion, the acetabular stress increased significantly at 100 degrees flexion, but the displacement did not improve much. However, there was no noticeable stress increase in the corresponding femoral head, and the stress on the top of the femoral head was significantly reduced than before with the rise of flexion angle

Distribution of simulated CCP on the acetabulum and corresponding femoral head for different abduction angles of the hip were depicted in Figs. 6, 7, 8. During the process of CR, the CCP distributed as butterfly-shaped, surrounding the U-shaped notch of the acetabulum and the femoral head. The contact area between the acetabular and the femoral head was larger on anterior and posterior than superior area, and with the increase of abduction angle, CCP became more and more uniform, suggesting that reduction was stable. However, extreme abduction angle (such as 80°) would significantly increase CCP on acetabulum in both front and behind, and both the corresponding anteromedial and lateral stress of the femoral head would substantially increase.

We also tried to simulate the reduction of the affected side, and investigated CCP’s change with difference flexion angles when the abduction is fixed in at the angle of 65°. Due to the narrow and long opening in the upper part of the acetabulum on the affected side, when the femoral head was repositioned, only the anterior and posterior edge of the acetabulum contacted with the femoral head with a small area, so the local stress and deformation of the acetabulum were relatively large. The direction and contact area between the femoral head and the acetabulum were different with different flexion angle, and the CCP would change accordingly. Compared with 90 degrees flexion, the acetabular stress increased significantly at 100 degrees flexion, but the displacement did not improve much. However, there was no noticeable stress increase in the corresponding femoral head, and the stress on the top of the femoral head was significantly reduced than before with the rise of flexion angle (Fig. 8).

## Discussion

This study is the first to investigate CCP in DDH patients during CR and explore the distribution of CCP with different abduction and flexion angles. During CR, the gravity of the lower extremities was offset by the plaster. Thus, the force maintaining the reduction was only the tension of muscles and force exerted by the surgeon during the reduction.

In case of complete hip dislocation, femoral head and acetabulum grow noninteractively, yielding a dysmorphia head and a shallow acetabulum (Fig. 5). Other researches have indicated that in case of lateral hip subluxation, the pressure on the femoral head concentrates in the medial aspect of the femoral head as the hip hinges along the edge of the acetabulum. Likewise, concentric pressure on the acetabular floor is reduced while it is increased along the lateral border. Then, the lateral aspect of the femoral head continues to grow and thus flattens the head. The acetabular growth cartilage fills the acetabular floor and arrests its lateral growth, forming a progressively shallower and more oblique acetabulum [11]. Liqun Duan et al. [13] established healthy children hip model, including complete cartilage and bone in hip joint. By applying FEA, they found that the cartilage in femoral head was thicker in the center and thinner in periphery, whereas the acetabular cartilage was the opposite. Shefelbine et al. [14] found that under loading conditions of the dysplastic hip, the octahedral shear stress was much more obvious on the medial side than the lateral, which promoted growth on the medial side and resulted in coxa valga. Shefelbine [15] also used FEA to compare the morphology between the normal side and affected side of the proximal femur, and concluded that the stress distribution of the femoral head epiphysis in DDH patients was significantly different from that in normal population, which contributes to the abnormal morphology of the femoral head. Recently, Vafaeian et al. [7] reported hip joint contact pressure distribution during Pavlik Harness (PH) treatment of infant. It turned out that PH position generates a horseshoe-shaped articular contact area involving most of the acetabulum but relatively sparing the superolateral portion, and a rapid increase in pressure with increasing leg abduction in harness may result in AVN.

Although we did not catch the finding of coxa valga of proximal femur from our 3-D model, we did find that the structure of the femoral head between two sides had a significant difference. Through the construction of the cartilage model of hip, it perfectly showed us the “flat-shaped” deformity of the affected side as compared with the “cup-shaped” acetabulum of the normal side, which to some extent explained why a severe dislocation hip was tough to reduce (Fig. 5).

By increasing the abduction angles, the simulated CCP demonstrates a “butterfly-shaped” distribution around the U-shaped notch of the acetabulum, due to more pressure absorbed in the anterior and superior acetabulum than the superior portion. Under that condition, it might deepen the acetabulum to guarantee stability during CR. This deepening may become morphologically permanent if femoral head position and pressure on the acetabulum are preserved during continuing growth of the cartilage [16]. Acetabular index (AI) was an important and most commonly used parameter to assess acetabular dysplasia. Li Y et al. [17] retrospectively compared AI, centre-edge angle of Wiberg (CEA), Reimer’s index and centre-head distance discrepancy over time among groups divided by final outcomes. According to their results, AI was the best post-surgical predictor. If AI > 28° for 1 year following CR or AI > 25° for two to 4 years after CR, the secondary surgery was warranted. Shin et al. [18] also recommended secondary surgery, if AI > 32° and CEA < 14° when patients older than 3 years. According to our results, the muscle force pushed the femoral head forward on Y-axis. Because the contact area between the femoral head and the superior acetabulum was small, on the Z-axis, the contact stress was minor between the acetabulum and the femoral head. Thus, during CR, inadequate pressure leads to minor stimulating effect, which explained the phenomenon of lack of improvement on the top of the acetabulum deformity after CR (Supplementary Fig. 3). It also explained why the AI is a good marker for failure of the CR.

For the late presenting or diagnosed patients, Nikolaos G et al. [19] reported a modified Hoffmann-Daimler functional method. By placing the dislocated hip in flexion (100°-120°), it allows the femoral head to gradually move into the acetabulum by the redirected action of the adductor and the flexor muscles followed by use of abduction splint (abduction angle 90°). It demonstrated satisfying outcomes with high success rate and low AVN rate among 69 patients (95 hips) with average of 11.5 years follow-up time. The acetabular index will be corrected as a result of the mechanical forces placed on the acetabulum. Some researchers [20] believe that extreme flexion angle (i.e.,> 120°) during CR could produce a femoral nerve palsy as the nerve could be compressed by the diapers between the thigh and abdomen. Hyperflexion may also cause the femoral head to dislocate inferiorly. Alternatively, inadequate flexion (i.e., < 90°) will fail to reduce the hip. In our study, the pressure against the femoral head decreased with the flexion angle changing from 90° to 100°, without apparent displacement of acetabulum (Fig. 8), suggesting that a better success rate of CR can be achieved by increasing the flexion angle appropriately.

AVN is a common and severe complication after the DDH treatment, occurring as high as in 60% of patients after CR [21]. The most common cause is the immobilization in a position that places excessive pressure on the femoral head. Thus, Ramsey et al. [22] recommended creating a “safe zone” to prevent AVN. In certain situation, an adductor tenotomy will increase the safe zone by allowing a wider range of abduction. However, extreme abduction should never be used because this has been shown to cause AVN. The relationship between hip abduction and blood-flow velocity in the femoral head has been established with Doppler ultrasound. In normal volunteers, the blood flow in femoral head drops significantly when abduction angle increases: with their hips in neutral position, mean blood flow was 13 cm/sec; at 30 degrees of abduction, it was 10.3 cm/sec; and at 45 degrees, 3.8 cm/sec [23]. The incidence of AVN varies widely (0 ~ 92.4%), mainly due to the lack of unified diagnostic criteria and some different understandings. Bradley et al. [24] conducted a retrospective study on AVN incidence after CR, and it was 10% among 441 DDH children (538 hips) on an average follow-up time of 7.6 years.

In our study, among the simulated five muscles, adductor magnus and adductor longus have the most potent restrictive effect on the abduction of the hip joint. Therefore, cutting adductor longus or adductor magnus during surgery can increase the abduction angle and maintain a stable reduction. As seen from Fig. 6, when the hip was flexed at 90° and abducted at 45°, the stress was mainly concentrated posteriorly. In this case, the contact area between the acetabulum and the femoral head was small, and the reduction was unstable. When the abduction angle was increased to 65°, the stress on the anterior area increased correspondingly, but the stress on the posterior had not increased significantly, which could result in more stable reduction, and distribute the pressure over the contact area more uniformly between the femoral head and the u-shaped notch, without excessive pressure on the femoral head. However, when the abduction of the hip was increased to 80°, CCP on the anterior and posteromedial acetabulum increased significantly, and the corresponding anterolateral and lateral stresses of the femoral head increased significantly, which would undoubtedly increase the pressure on the supportive and epiphyseal arteries of the femoral head, which is very important for the growth and development of femoral head in childhood.

There were multiple necessary improvements and limitations in the model. 1) Since anesthesia is administrated, it may cause of data collection problem, because 3D MRI will take much longer time than the usual one. 2) The unaffected hip was constructed to simulate the distribution of CCP during CR at various abduction angles and flexion angles, which might be incomparable to the affected side. 3) Only adductor muscle and operator force were applied to simulate the force of the joint, which ignores the possibility of soft tissues involvement in the hip joint obstructing reduction, as well as the effect of hip capsule and surrounding ligaments on CCP. Using unaffected side hip may help to control for this involvement. 4) There is no quantitative validation of the muscle forces and CCP, which is currently unsolvable, since in vivo measurements are not possible due to technological challenges and ethical concerns. In the future work, we will continue to conduct simulation analysis on the affected side and load more boundary conditions, or using baroreceptor to simulate the biomechanical changes in the process of CR from the most realistic perspective.

## Conclusion

This is the first study of applying FEA to simulate CCP changes during CR of DDH and analyze its biomechanical changes. Severe dislocation (IHDI grade III and IV) in children have shown high mismatch between the femoral head and the acetabulum, so the reduction was steep. We observed a “butterfly-shaped” CCP distribution surrounding the U-shaped acetabular notch, with relative sparing of the anterior and posterior of acetabular. We also observed a more even distribution of CCP and a rapid increase in pressures with increased leg abduction angle and a modest increase in pressures with increased leg flexion angle. We believe that may be the reason that the extreme abduction would maintain stability but increase the risk of AVN and the appropriately increasing flexion angle might lead to satisfying outcomes. A less contact of the acetabular roof was depicted during CR. Thus, the stress-induced growth was weak, and the deformity of the acetabular roof cannot be significantly corrected, which may explain why AI was the best marker to predict the risk for secondary surgery.

## Supplementary information

**Additional files 1.**

## Data Availability

The datasets used and/or analyzed during the current study are available from the corresponding author on reasonable request.

## Abbreviations

DDH: Developmental dysplasia of the hip
CR: Closed reduction
CCP: Cartilage contact pressure
AVN: Avascular necrosis
FEA: Finite element analysis
IHDI: International Hip Dysplasia Institute
AI: Acetabular index
PCSA: Physiological cross-sectional area
PH: Pavlik Harness
CEA: Centre-edge angle
