# Supplementary figures and images for: Contact pressure distribution of the hip joint during closed reduction of developmental dysplasia of the hip: a patient-specific finite element analysis
Source: BMC Musculoskelet Disord. 2020 Sep 8;21:600. doi: 10.1186/s12891-020-03602-w (PMC7487652; doi:10.1186/s12891-020-03602-w)

# Supplementary Figures

Fig.1


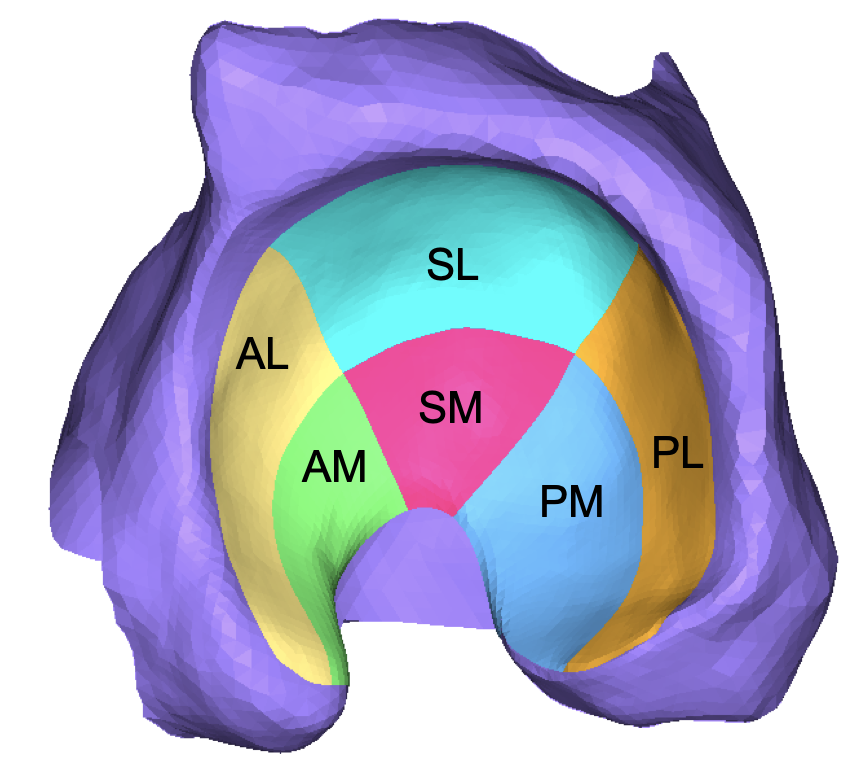

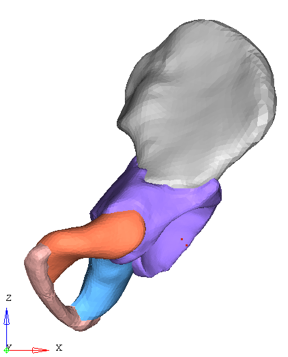


Fig.2


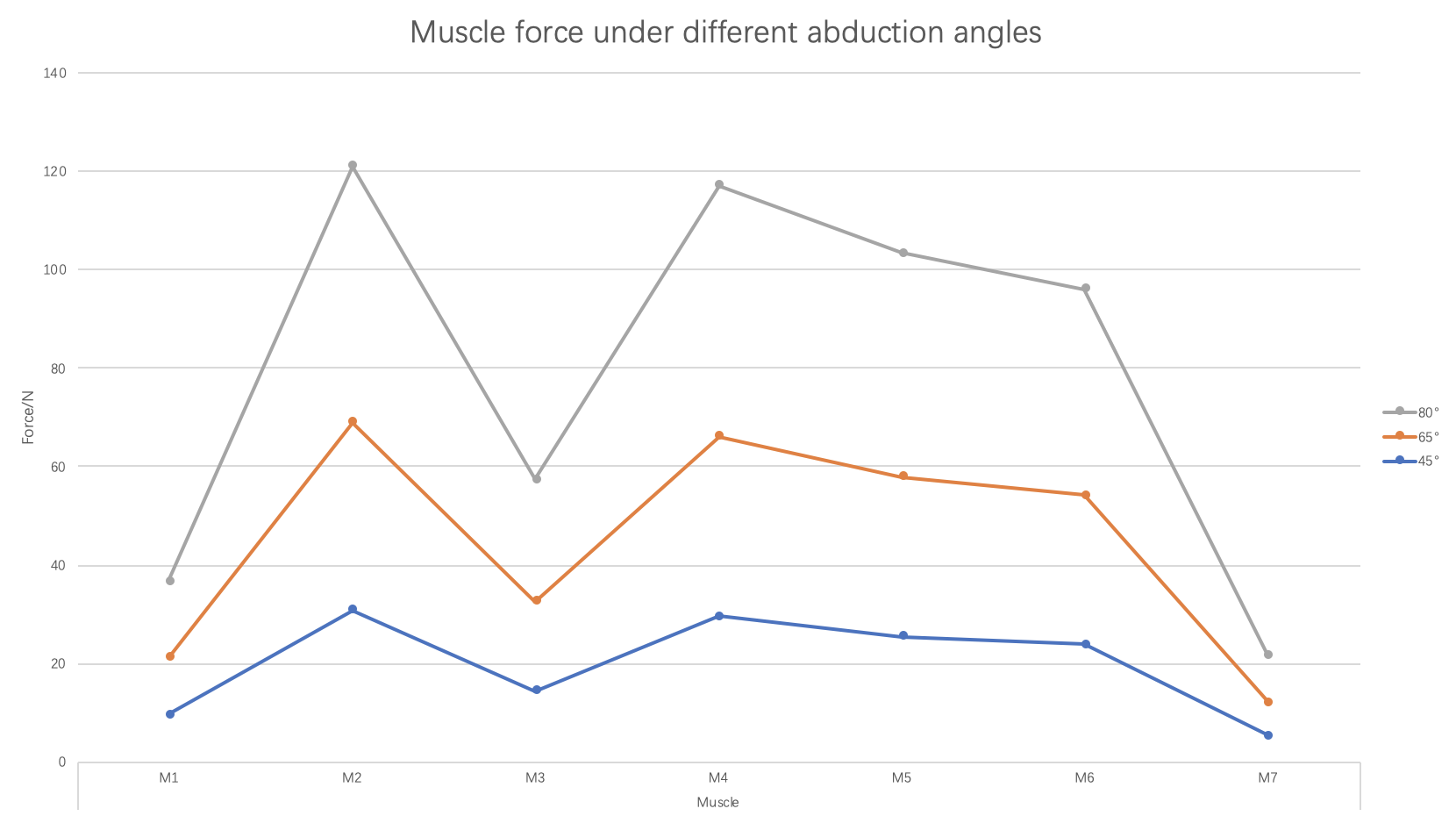


Fig.3


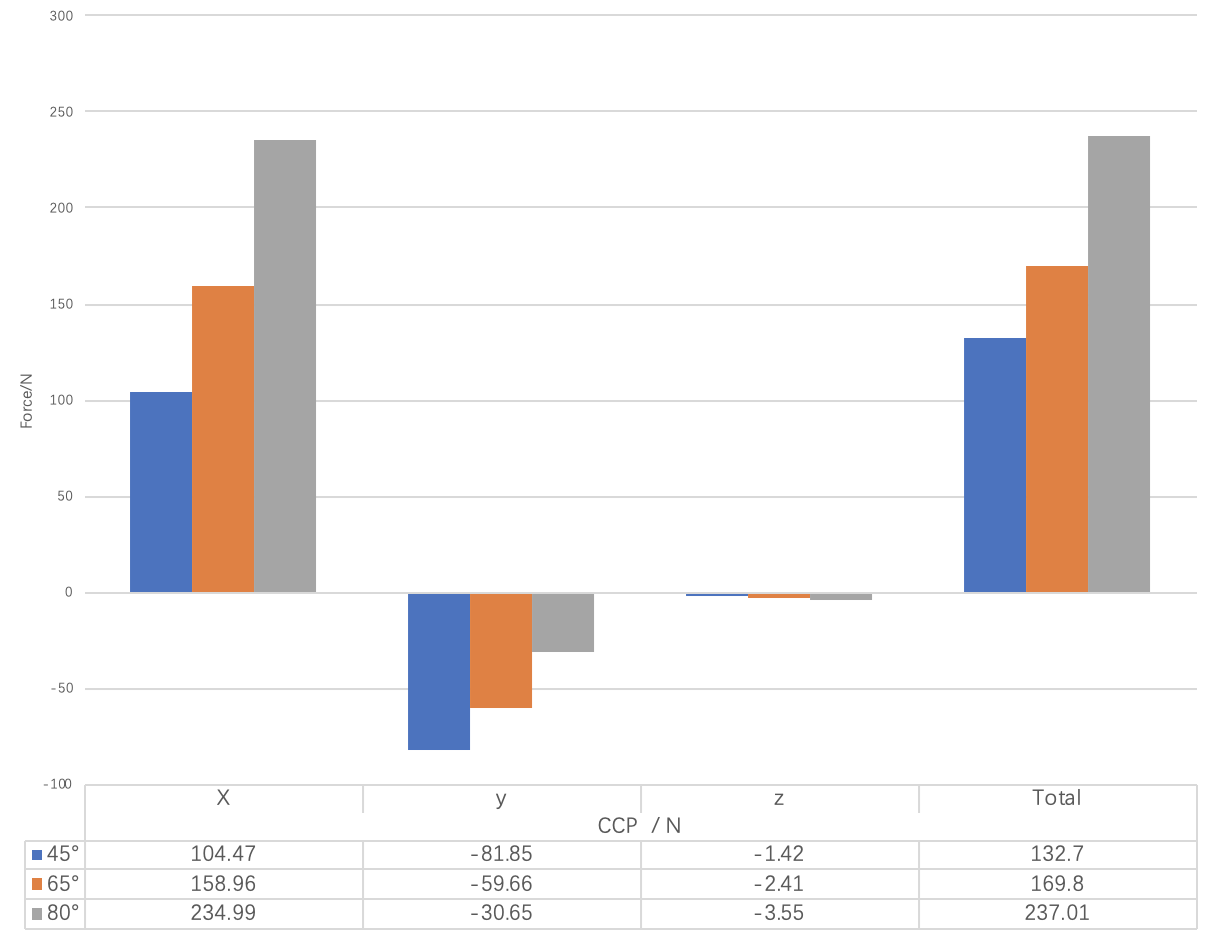

Supplement: Supplementary file 1 — Additional files 1. [file 12891_2020_3602_MOESM1_ESM.docx]
